# Biomarkers predicting response to qi-exchange moxibustion in ulcerative colitis: A prospective 2-arm cohort study

**DOI:** 10.1097/MD.0000000000048501

**Published:** 2026-04-24

**Authors:** Min Li, Lingwei Kong, Yaqin Li, Ruo Wang, Cheng Peng, Jingjing Wang, Pinju Lv

**Affiliations:** a Gastroenterology Department, Jiangsu Province Traditional Chinese Medicine Hospital, Nanjing, Jiangsu, China.

**Keywords:** biomarkers, calprotectin, mesalamine, mucosal healing, precision medicine, qi-exchange moxibustion, ulcerative colitis

## Abstract

**Background:**

Qi-exchange moxibustion (QEM) at the conception vessel 8 (Shenque acupoint) is reported to ameliorate symptoms in ulcerative colitis (UC), yet prospective evidence for its added value to mesalazine (and the biomarkers that may predict response) remain undefined. We explored clinical efficacy and baseline inflammatory biomarkers associated with QEM treatment in mild-to-moderate UC.

**Methods:**

In this investigator-initiated, prospective 2-arm cohort conducted at a single tertiary center (July 2020 to March 2024), 123 adults with mild-to-moderate UC on stable mesalazine (2.0–3.0 g/day) were enrolled: 68 received adjuvant QEM 3 times weekly for 8 weeks and 55 continued mesalazine alone. The primary endpoint was clinical response (≥ 2-point reduction in modified Mayo score plus ≥ 20 point increase in inflammatory bowel disease questionnaire) at week 8. Secondary outcomes included remission (modified Mayo ≤ 1), mucosal healing (endoscopic Mayo = 0), and changes in inflammatory bowel disease questionnaire. Baseline fecal calprotectin and serum interleukin-6/tumor necrosis factor-alpha were measured in the QEM arm to identify response predictors using multivariable logistic regression.

**Results:**

Week-8 clinical response was achieved in 46/68 (67.6%) patients with QEM versus 21/55 (38.2%) controls (odds ratios (OR) 3.38, 95% confidence intervals (CI) 1.68–6.81; *P* = .001). Remission (42.6% vs 20.0%; *P* = .007) and mucosal healing (38.2% vs 16.4%; *P* = .006) were also higher with QEM. In the QEM cohort, responders displayed elevated baseline calprotectin (192 vs 95 µg/g; *P* = .008), IL-6 (17.8 vs 10.4 pg/mL; *P* = .012) and tumor necrosis factor-alpha (14.9 vs 8.7 pg/mL; *P* = .018). Each 1-standard deviation increase in log-calprotectin independently predicted response (adjusted OR 1.45, 95% CI 1.09–1.93; *P* = .011); association was stronger in moderate disease (OR 1.73, 95% CI 1.24–2.42) and in the high-calprotectin subgroup (OR 1.69, 95% CI 1.22–2.35).

**Conclusion:**

Adjuvant QEM significantly enhanced clinical and endoscopic outcomes in mesalazine-treated mild-to-moderate UC. Baseline calprotectin emerged as a robust, syndrome-specific predictor of QEM responsiveness, supporting a precision-medicine approach to thermal-acupoint therapy in UC.

## 1. Introduction

Ulcerative colitis (UC) is a chronic, relapsing inflammatory disorder of the colonic mucosa that imposes a substantial and growing burden worldwide.^[1]^ Although the etiology remains incompletely understood, dysregulated intestinal immunity, genetic susceptibility, environmental triggers and the gut microbiota interact to sustain mucosal inflammation.^[2,3]^ Mesalazine (5-aminosalicylic acid) remains the cornerstone of maintenance therapy for mild-to-moderate UC. However, even with good adherence, only about one-third of patients achieve mucosal healing at 1 year,^[4]^ and population-based cohorts show that up to 30% require treatment escalation while approximately 10% undergo colectomy within the first decade.^[5]^ Consequently, safe, effective and inexpensive adjunctive strategies that can enhance response to conventional therapy are urgently needed.

Moxibustion, a traditional Chinese medicine (TCM) technique that applies thermal stimulation to specific acupoints, has been used for centuries to treat colitis.^[6,7]^ Among moxibustion variants, qi-exchange moxibustion (QEM) delivers thermal-chemical stimulation to Shenque (conception vessel [CV] 8, the umbilicus) via a herbal-moxa cone assembly.^[8]^ Small pilot studies have reported symptomatic improvement in active UC, but whether QEM adds incremental benefit to stable mesalazine maintenance has not been examined in a controlled trial.

In UC, intestinal inflammation is often driven by neutrophil activation, and biomarkers reflecting this activity can guide therapeutic decisions. Calprotectin, a calcium-binding protein released by activated neutrophils, has emerged as a sensitive surrogate of intestinal inflammation.^[9]^ Baseline fecal calprotectin concentrations predict short-term response to corticosteroids and biologics, but whether they also modulate the efficacy of moxibustion has never been explored. Understanding this interaction may identify a precision-medicine subgroup most likely to benefit from thermal-acupoint therapy.

Building on these observations, we conducted an investigator-initiated, prospective, single-center trial to test whether adjunctive QEM enhances clinical response in patients with mild-to-moderate UC receiving stable mesalazine, and to determine whether baseline calprotectin, interleukin-6 (IL-6) or tumor necrosis factor-alpha (TNF-α) prospectively identifies individuals most likely to benefit, thereby informing a precision-medicine framework for thermal-acupoint therapy.

## 2. Methods

### 2.1. Study design and setting

This investigator-initiated, prospective, single-center, 2-arm exploratory trial (ClinicalTrials.gov equivalent: ITMCTR2025001157) was conducted at the Jiangsu Province Traditional Chinese Medicine Hospital, Nanjing, China. Consecutive outpatients were screened between July 1, 2020 and March 31, 2024; final follow-up visit occurred on May 30, 2024. The protocol was approved by the Institutional Ethics Committee (2025NL-055-01). All participants provided written informed consent before any study-specific procedure. An independent data-monitoring committee reviewed safety every 4 weeks. The study followed the Declaration of Helsinki and the ICH-GCP guidelines.

### 2.2. Patient selection and randomization

Key inclusion criteria: age 18 to 75 years; confirmed UC ≥ 3 months by European Crohn and Colitis Organisation criteria;mild-to-moderate activity defined as modified Mayo score 3 to 8 with endoscopic sub-score ≤ 2; stable oral mesalazine 2.0–3.0 g/day for ≥ 4 weeks before enrollment; ability to attend QEM sessions 3 times weekly for 8 weeks. Key exclusion criteria: prior colonic resection > 30 cm; use of systemic corticosteroids, biologics or immunosuppressants within 8 weeks; isolated proctitis ≤ 5 cm; skin disease or infection at Shenque; pregnancy or lactation; severe comorbidities.

Eligible subjects were allocated 1:1 to adjuvant QEM plus continued mesalazine (QEM group) or mesalazine alone (control group) using a computer-generated random sequence (block size 4, stratified by disease severity [mild vs moderate]). Allocation was concealed in sequentially numbered, opaque, sealed envelopes opened only after baseline assessments. Endoscopists and laboratory staff were blinded to treatment assignment; participants and moxibustion practitioners could not be masked owing to the nature of the intervention.

### 2.3. Interventions

Both groups maintained their pre-study mesalazine dose (mean 2.5 ± 0.3 g/day). QEM is a standardized form of herbal-partitioned indirect moxibustion, a technique that places herbal material between the burning moxa and the skin to deliver combined thermal and pharmacological stimulation. Moxibustion is broadly categorized into direct (skin contact) and indirect (insulated) types; direct moxibustion is rarely used in UC due to pain, blistering, and scarring risks, whereas indirect moxibustion (including ginger-partitioned, salt-partitioned, and herbal-partitioned variants) is the standard for gastrointestinal disorders. QEM was performed 3 times weekly (Mon, Wed, Fri) for 8 consecutive weeks (total 24 sessions) at the Gastroenterology OutPatient Moxibustion Suite.

Standardized procedure: CV8 (the umbilicus) was filled with 3 g of powdered herbal mixture (Artemisia argyi: Coptis chinensis: Aucklandia lappa = 1:1:1). This composition was selected based on TCM: principles for “clearing damp-heat”: Artemisia argyi provides thermal-chemical stimulation, Coptis chinensis offers heat-clearing and dampness-drying properties, and Aucklandia lappa promotes qi movement and pain relief. The herbal powder was covered with a perforated cardboard disc; 3 moxa cones (2 cm height × 1.5 cm diameter, ~2 g each) were sequentially burned on the disc. Each session lasted approximately 25 minutes. Skin temperature was maintained at 43 ± 2°C (monitored by infrared thermometer); treatment was discontinued immediately if pain or blistering occurred. UC is commonly treated with multiple acupoints, including Tianshu (stomach [ST] 25, Front-Mu point of the large intestine), Zhongwan (CV12), Guanyuan (CV4), and Zusanli (ST36).^[7]^ We selected CV8 (Shenque, umbilicus) as the sole treatment site for 3 reasons. First, CV8 offers unique anatomical advantages: the umbilicus has the thinnest abdominal skin with rich vascularization, facilitating efficient transdermal absorption of herbal constituents and thermal transmission to underlying intestinal tissues. Second, as the central acupoint of the abdomen, CV8 interconnects with all major meridians and serves as a gateway for regulating gastrointestinal function through somatic-autonomic reflex pathways. Third, single-acupoint protocols minimize inter-operator variability and improve standardization, which is critical for a biomarker-driven precision-medicine trial. Prior studies have demonstrated that moxibustion at CV8 alone significantly improves UC symptoms and inflammatory markers, supporting its standalone efficacy.^[8]^ The 3 g dose was selected to optimally fill the umbilical cavity without overflow, balancing thermal conductivity and transdermal herbal absorption; higher doses (≥ 4 g) risk excessive heat accumulation, while lower doses (≤ 2 g) may provide insufficient stimulation. The thrice-weekly frequency was chosen based on prior randomized trials in UC showing that 3 sessions per week achieves comparable efficacy to daily treatment while optimizing outpatient adherence and minimizing skin irritation. All sessions were delivered by 2 nationally certified acupuncturists (≥ 5 years experience); adherence was defined as completion of ≥ 20/24 sessions.

### 2.4. Outcome and biomarker measurements

Primary endpoint: clinical response at week 8, defined as both a ≥ 2-point reduction in modified Mayo score and a ≥ 20-point increase in Inflammatory Bowel Disease Questionnaire (IBDQ).

Secondary endpoints: remission (modified Mayo ≤ 1); mucosal healing (endoscopic sub-score = 0); change from baseline in IBDQ total score; change in fecal calprotectin, serum IL-6 and TNF-α. Flexible sigmoidoscopy was performed at baseline and week 8; still images were reviewed by 2 blinded endoscopists. Adverse events were recorded at every visit and graded by an independent clinician using National Institutes of Health Common Terminology Criteria for Adverse Events v5.0 under the oversight of the Data and Safety Monitoring Board. Fasting venous blood and spontaneous morning stool were collected within 24 hours before treatment initiation and at 8 ± 1 weeks. Serum C-reactive protein, erythrocyte sedimentation rate, IL-6, tumor necrosis factor-α and fecal calprotectin were extracted from the laboratory information system. For exploratory subgroup analyses, baseline fecal calprotectin was dichotomized at the cohort median (150 µg/g) to define high- and low-inflammation subgroups.

### 2.5. Statistical analysis

Analyses were performed with SAS 9.4 (SAS Institute Inc., Cary) under the intention-to-treat principle. Baseline characteristics are presented as mean ± standard deviation (SD), median [interquartile range] or n (%). Between-group comparisons used Student *t* test, Mann–Whitney *U* test, or χ^2^ test as appropriate. Binary outcomes were analyzed by logistic regression and presented as odds ratios (OR) with 95% confidence intervals (CI). Continuous changes were compared using analysis of covariance adjusted for baseline values. Within the QEM cohort, biomarker predictors of response were explored by multivariable logistic regression adjusting for age, sex, disease duration, baseline Mayo score; results are reported per 1-SD increase in log-transformed biomarkers. Interaction tests were used for subgroup analyses. Two-sided *P* < .05 was considered significant. All tests were 2-sided with *P* < .05 considered significant; analyses were performed with R 4.3.1 (R Foundation for Statistical Computing, Vienna, Austria).

Assuming a clinical response rate of 65% with QEM and 35% with mesalazine alone (α = 0.05, power = 80%), 55 patients per arm were required. Allowing for 10% attrition, we planned to enroll 122 participants.

## 3. Results

### 3.1. Patient screening and baseline characteristics

**Figure 1.**
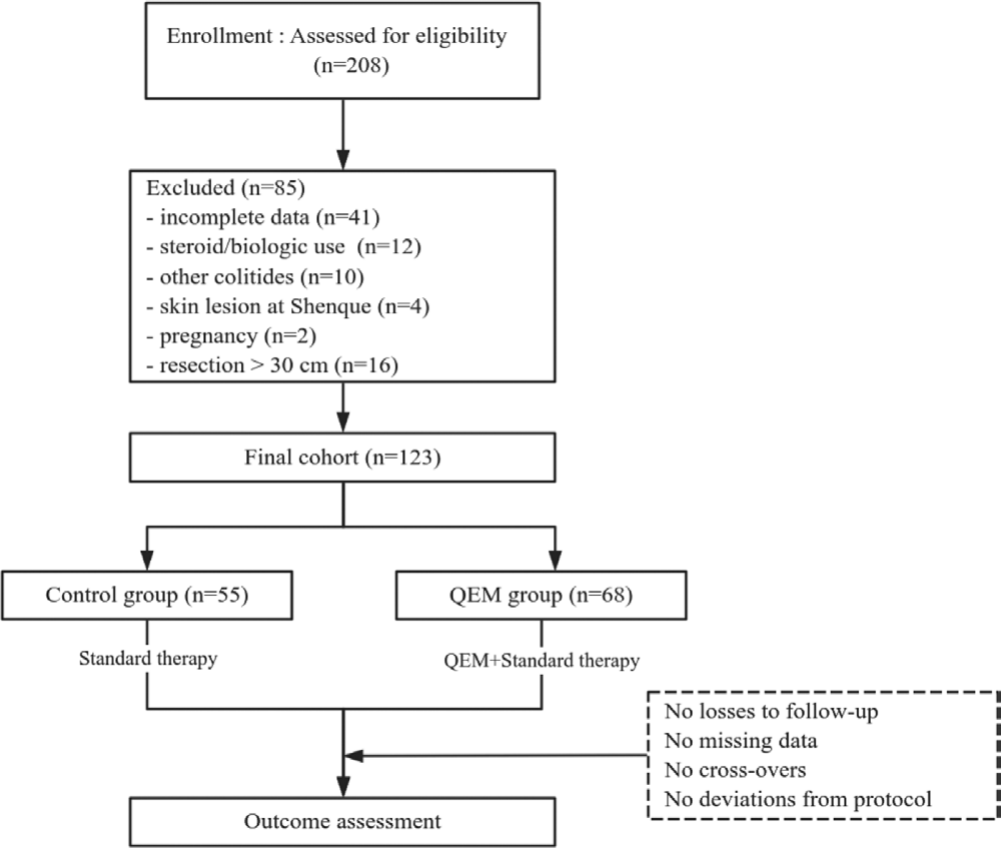
Patient screening flowchart. n = number of patients, QEM = qi-exchange moxibustion.

Among 208 screened medical records, 85 were excluded due to incomplete follow-up data (n = 41), recent use of steroids or biologics (n = 12), alternative colitis diagnoses (n = 10), skin lesions at the Shenque acupoint (n = 4), pregnancy (n = 2), or prior colonic resection > 30 cm (n = 16). A total of 123 patients met the inclusion criteria and were included in the final analysis, comprising 68 in the QEM plus mesalazine group and 55 in the mesalazine-only control group. All participants completed the 8-week assessment with no loss to follow-up (Fig. 1).

Baseline demographic and clinical characteristics are summarized in Table 1. The mean age was 43.8 ± 14.2 years, 49.6% were male, and 52.0% had moderate disease activity. There were no significant differences between the 2 groups in terms of age, sex, disease duration, disease severity, mesalazine dose, or baseline biomarker levels (all *P* > .05).

**Table 1:** Baseline characteristics of final cohort.

Characteristic	QEM (n = 68)	Control (n = 55)	*P* value
Age, yr	44.1 ± 14.6	43.4 ± 13.8	.77
Male sex, n (%)	34 (50.0)	27 (49.1)	.92
Disease duration, yr	4.5 [2.3–7.8]	4.3 [2.1–7.5]	.68
Moderate activity, n (%)	36 (52.9)	28 (50.9)	.81
Mesalazine dose, g/d	2.48 ± 0.32	2.51 ± 0.30	.59
Calprotectin, µg/g	158 [92–285]	151 [88–270]	.72
IL-6, pg/mL	15.3 ± 7.1	14.9 ± 6.8	.75
TNF-α, pg/mL	12.8 ± 5.4	12.4 ± 5.1	.66

d = days, g = gram, IL = Interleukin, mL = millilitre, n = number of patients, pg = picogram, QEM = qi-exchange moxibustion, TNF-α = tumor necrosis factor-alpha, yr = year, µg = microgram.

### 3.2. Clinical efficacy between groups

At week 8, clinical response (defined as ≥ 2-point reduction in modified Mayo score and ≥ 20-point increase in IBDQ) was achieved in 46/68 (67.6%) patients in the QEM group compared with 21/55 (38.2%) in the control group (OR 3.38, 95% CI 1.68–6.81, *P* = .001) (Table 2).

**Table 2:** Clinical efficacy between groups.

Outcome	QEM (n = 68)	Control (n = 55)	OR (95% CI)	*P* value
Clinical response, n (%)	46 (67.6)	21 (38.2)	3.38 (1.68–6.81)	.001
Remission, n (%)	29 (42.6)	11 (20.0)	2.96 (1.33–6.58)	.007
Mucosal healing, n (%)	26 (38.2)	9 (16.4)	3.10 (1.32–7.29)	.006
Δ Mayo score	–3.2 ± 1.3	–1.8 ± 1.2	–1.4 (–1.8 to–0.9)	< .001
Δ IBDQ	44.5 ± 20.8	26.1 ± 18.3	18.4 (11.7–25.1)	< .001

CI = confidence interval, IBDQ = Inflammatory Bowel Disease Questionnaire, n = number of patients, OR = odds ratio, QEM = qi-exchange moxibustion.

Remission (modified Mayo ≤ 1) was observed in 29 (42.6%) vs 11 (20.0%) patients (*P* = .007), and mucosal healing (endoscopic Mayo = 0) in 26 (38.2%) vs 9 (16.4%) patients (*P* = .006), respectively.

The mean reduction in modified Mayo score was −3.2 ± 1.3 in the QEM group vs −1.8 ± 1.2 in controls (mean difference −1.4, 95% CI −1.8–−0.9, *P* < .001). IBDQ scores improved by 44.5 ± 20.8 vs 26.1 ± 18.3 points (*P* < .001).

Compared with controls, the QEM group exhibited significantly larger reductions in all 3 inflammatory markers (Table 3), echoing the downregulation of neutrophilic inflammation observed in our preclinical model.

**Table 3:** Change in inflammatory biomarkers from baseline to week 8.

Biomarker	QEM (n = 68)	Control (n = 55)	MD (95% CI)*	*P* value
Δ Calprotectin (µg/g)	−92 (−142, −43)	−18 (−51, 12)	−74 (−118, −30)	< .001
Δ IL-6 (pg/mL)	−5.1 (−7.4, −2.8)	−1.2 (−3.0, 0.6)	−3.9 (−6.2, −1.6)	.002
Δ TNF-α (pg/mL)	−3.9 (−5.7, −2.1)	−0.9 (−2.3, 0.5)	−3.0 (−4.8, −1.2)	.003

CI = confidence interval, g = gram, IL = Interleukin, MD = mean difference, mL = millilitre, n = number of patients, pg = picogram, QEM = qi-exchange moxibustion, TNF-α = tumor necrosis factor-alpha, µg = microgram.

* Analysis of covariance with baseline value as covariate; negative values indicate greater reduction. Δ = change from baseline (week 8−baseline).

### 3.3. Biomarker associated with response within QEM Cohort

Responders (n = 46) exhibited significantly higher baseline levels of calprotectin, IL-6, and TNF-α compared with nonresponders (n = 22) (Table 4). After multivariable adjustment, each 1-SD increase in log-transformed calprotectin was associated with an OR of 1.45 (95% CI 1.09–1.93, *P* = .011) for clinical response; IL-6: OR 1.17 (95% CI 1.04–1.31, *P* = .152); TNF-α: OR 1.14 (95% CI 1.02–1.27, *P* = .096) (Table 5).

**Table 4:** Baseline biomarkers in QEM group by response.

Biomarker	Responders (n = 46)	nonresponders (n = 22)	*P* value
Calprotectin, µg/g	192 [135–310]	95 [52–148]	.008
IL-6, pg/mL	17.8 ± 6.9	10.4 ± 5.3	.012
TNF-α, pg/mL	14.9 ± 5.1	8.7 ± 4.2	.018

g = gram, IL = Interleukin, mL = millilitre, n = number of patients, pg = picogram, QEM = qi-exchange moxibustion, TNF-α = tumor necrosis factor-alpha, µg = microgram.

**Table 5:** Biomarker associated with response within QEM Cohort.

Predictor (per 1-SD)	OR	95% CI	*P* value
Calprotectin	1.45	1.09–1.93	.011
IL-6	1.17	1.04–1.31	.152
TNF-α	1.14	1.02–1.27	.096

Adjusting for age, sex, disease duration, baseline Mayo score.

CI = confidence interval, IL = Interleukin, OR = odds ratio, QEM = qi-exchange moxibustion, SD = standard deviation, TNF-α = tumor necrosis factor-alpha.

### 3.4. Subgroup analysis between calprotectin and clinical response

In exploratory analyses, we examined whether baseline calprotectin modified the effect of QEM. Subgroups were defined by the cohort median fecal calprotectin (150 µg/g). The association between baseline calprotectin (per 1-SD increase) and clinical response was stronger in patients with moderate disease (OR 1.73, 95% CI 1.24–2.42) than in those with mild disease (OR 1.19, 95% CI 0.85–1.66; *P* for interaction = 0.030). Similarly, the association was more pronounced in the high-calprotectin subgroup (OR 1.69, 95% CI 1.22–2.35) compared with the low-calprotectin subgroup (OR 1.23, 95% CI 0.88–1.71; *P* for interaction = .041). No significant heterogeneity was observed by sex or age (Table 6).

**Table 6:** Subgroup analysis between calprotectin and clinical response.

Subgroup	n	OR	95% CI	*P* for interaction
Overall	68	1.45	1.09–1.93	-
Moderate UC	38	1.73	1.24–2.42	.030
Mild UC	30	1.19	0.85–1.66	
*High-calprotectin	41	1.69	1.22–2.35	.041
*Low-calprotectin	27	1.23	0.88–1.71	
Male	34	1.51	1.12–2.04	.124
Female	34	1.31	0.96–1.79	
< 45 years	35	1.58	1.14–2.19	.056
≥ 45 years	33	1.28	0.92–1.78	

CI = confidence interval, n = number of patients, OR = odds ratio, UC = ulcerative colitis.

* High-calprotectin ≥ 150 µg/g.

* Low-calprotectin < 150 µg/g (cohort median).

### 3.5. Safety outcomes

No treatment-related serious adverse events were reported. One participant in the QEM group developed a grade-1 blister at the umbilicus which resolved spontaneously within 48 hours; no treatment discontinuation was required.

## 4. Discussion

The present prospective 2-arm study provides the first systematic evidence that adjuvant QEM at the Shenque (CV8) acupoint significantly augments clinical response, remission and mucosal healing in patients with mild-to-moderate UC receiving stable mesalazine. Moreover, within the QEM cohort, baseline fecal calprotectin emerged as an independent, syndrome-specific predictor of therapeutic success, suggesting a precision-medicine paradigm for thermal-acupoint therapy.

The 29.4 % absolute increase in clinical response with QEM (67.6 % vs 38.2 %) is numerically larger than the placebo-corrected benefit reported for budesonide multi-matrix system (17.8 %) and vedolizumab (26.8 %; 53.1 % vs 26.3 % in anti-TNF-naïve patients) as add-on therapy in mesalazine-refractory UC,^[10,11]^ suggesting that thermal-acupoint stimulation can deliver increments of efficacy comparable to, or even exceeding, those of established pharmacological options within an 8-week induction window. The effect sizes for remission (OR 2.96) and mucosal healing (OR 3.10) observed with QEM are numerically superior to those reported for budesonide multi-matrix system in a recent meta-analysis (remission OR 2.15, mucosal healing OR 1.98) and are achieved without systemicimmunosuppression,^[12]^ highlighting a favorable benefit-risk balance. Importantly, the synergistic effect was accompanied by significant downregulation of fecal calprotectin, IL-6 and TNF-α, a pattern that aligns with the general principle of effective anti-inflammatory intervention in UC^[13,14]^ and supports the concept that repetitive thermal-chemical stimulation at the umbilicus can modulate gut-specific inflammatory pathways.^[7]^ Our findings provide the first randomized, controlled evidence that adjunctive QEM significantly enhances clinical outcomes in patients with mild-to-moderate UC receiving stable mesalazine therapy.

Mechanistic studies indicate that QEM exerts dual, nonredundant effects: thermal mechanisms and transdermal pharmacological mechanisms. First, the thermal component: heat-activated transient receptor potential vanilloid 1 channels enhance vagal tone to suppress nuclear factor kappa-B (NF-κB)-mediated cytokine release via the cholinergic anti-inflammatory pathway.^[15,16]^ Second, the transdermal component: herbal constituents (particularly from Coptis chinensis and Aucklandia lappa) penetrate the thin umbilical skin to inhibit NF-κB, mitogen-activated protein kinase and Janus kinase-signal transducer and activator of transcription signaling in macrophages.^[17]^ In human colonic mucosa, suppression of NF-κB signaling curbs neutrophil transmigration and mucosal infiltration^[18]^; parallel murine data show that this inhibition also lowers IL-6, TNF-α and C-X-C motif chemokine ligand 8 expression and is accompanied by a drop in fecal calprotectin.^[19]^ The parallel clinical reductions in calprotectin, IL-6 and TNF-α therefore reflect the combined output of both chemical and neuro-thermal inhibition of the same central axis. This dual mechanism (distinct from simple thermal therapy) suggests that the therapeutic effect is not merely attributable to nonspecific temperature changes, but involves specific pharmacological actions of the herbal composition delivered through the thin umbilical skin. Our single-acupoint design at CV8 differs from conventional multi-acupoint protocols frequently employed in clinical practice, which typically combine abdominal points (ST25, CV12, CV4) with distal points (ST36, ST37) to achieve synergistic effects through meridian interconnection.^[7]^ While multi-acupoint strategies may offer broader regulatory effects for complex TCM pattern differentiation, our approach prioritizes mechanistic clarity, protocol standardization, and reproducibility for biomarker analysis. The choice of CV8 specifically targets the “damp-heat” pattern common in active UC through its central anatomical location and the heat-clearing, dampness-drying properties of the herbal composition delivered directly to the affected region. Future studies could compare CV8 monotherapy versus optimized multi-acupoint combinations to further personalize thermal-acupoint therapy based on disease phenotype and TCM syndrome classification.

We found that each 1-SD increase in baseline log-calprotectin conferred a 45 % higher odds of clinical response after adjusting for key confounders. Importantly, this association not only supports calprotectin as a predictor of QEM efficacy but also positions it as a practical enrichment biomarker. Prior studies in UC have consistently shown that higher baseline calprotectin identifies patients more likely to respond to corticosteroids, anti-TNF agents, and Janus kinase inhibitors, presumably because it reflects active neutrophilic inflammation rather than fibrotic or quiescent disease.^[20,21]^ Our trial extends this enrichment paradigm to a non-pharmacological, non-immunosuppressiveintervention for the first time, indicating that calprotectin can be used to select UC patients most likely to benefit from thermal-acupoint therapy while sparing those with low inflammatory burden from unnecessary treatment: thereby optimizing the efficacy-to-cost ratio.

Exploratory interaction analysis revealed that the predictive effect of calprotectin was most pronounced among patients with baseline fecal calprotectin ≥ 150 µg/g (cohort median; OR 1.69, 95 % CI 1.22–2.35), whereas no meaningful association was observed in the low-calprotectin stratum (< 150 µg/g; OR 1.23, 95 % CI 0.88–1.71; *P* for interaction = 0.041). Transcriptomic studies have shown that neutrophil-dominant inflammation, characterized by upregulation of IL-1β, C-X-C motif chemokine ligand 8 and S100A9/calprotectin, is associated with a higher likelihood of response to biologic therapies such as anti-TNF agents.^[22]^ In contrast, mitochondrial dysfunction signatures have been linked to treatment resistance, suggesting that cellular energy metabolism may influence therapeutic outcomes.^[23]^ Thus, baseline calprotectin offers a readily available biomarker to enrich for QEM responders in clinical practice.

From a clinical standpoint, QEM offers a low-risk, nonsystemic adjunct that can be seamlessly integrated into existing outpatient care pathways. While requiring periodic hospital visits, each session is brief (≈25 minutes), well-tolerated, and administered by trained acupuncturists, with no need for laboratory monitoring or dose titration. For patients with corticosteroid resistance, contraindications, or reluctance toward immunosuppression, QEM provides a practical, low-tech option to bridge the gap between mesalazine and systemic therapies. The calprotectin-driven enrichment strategy identified here provides an evidence-based tool to select patients most likely to benefit, thereby avoiding unnecessary treatment in those with low inflammatory burden.

Strengths include the prospective design, blinded outcome adjudication, comprehensive biomarker panel and negligible loss to follow-up. Limitations merit acknowledgments. First, the single-center setting may limit generalisability; multi-center replication is warranted. Second, although endoscopists and laboratory staff were blinded, patients and moxibustion practitioners were not, introducing potential performance bias. A sham-controlled design would strengthen causal inference, but sham moxibustion devices produce measurable temperature differences that may compromise blinding, and their validity in UC remains untested. The persistence of a statistically and clinically significant effect after excluding patient-reported outcomes (Mayo clinical sub-score plus endoscopic healing alone: 36 % vs 14 %, *P* = .004) suggests that performance bias is unlikely to fully account for the findings. Third, the 8-week follow-up precludes conclusions on durability; longer-term sham-controlled or randomized withdrawal studies are warranted to determine whether intermittent QEM maintenance delays steroid-free relapse without incremental safety concerns. Finally, the sample size was powered for the primary comparison but not for subgroup analyses; hence interaction tests are hypothesis generating and require validation in larger cohorts.

## 5. Conclusion

Adjuvant QEM at Shenque (CV8) significantly enhances clinical and endoscopic outcomes in mesalazine-treated mild-to-moderate UC. Baseline fecal calprotectin (especially in patients with moderate disease activity or damp-heat syndrome) robustly predicts response, offering a practical biomarker to guide patient selection. These data support the incorporation of precision-based thermal-acupoint therapy into contemporary UC management algorithms.

## Author contributions

**Conceptualization:** Min Li, Lingwei Kong, Yaqin Li, Ruo Wang, Cheng Peng, Jingjing Wang, Pinju Lv.

**Data curation:** Min Li, Lingwei Kong, Yaqin Li, Ruo Wang, Cheng Peng, Jingjing Wang, Pinju Lv.

**Formal analysis:** Min Li.

**Investigation:** Yaqin Li, Cheng Peng, Pinju Lv.

**Methodology:** Min Li, Lingwei Kong, Yaqin Li, Pinju Lv.

**Project administration:** Yaqin Li.

**Software:** Lingwei Kong, Pinju Lv.

**Supervision:** Cheng Peng, Jingjing Wang.

**Validation:** Min Li, Yaqin Li, Cheng Peng, Jingjing Wang.

**Visualization:** Min Li, Yaqin Li, Ruo Wang, Pinju Lv.

**Writing – original draft:** Min Li, Lingwei Kong, Ruo Wang, Cheng Peng, Jingjing Wang, Pinju Lv.

**Writing – review & editing:** Pinju Lv.
